# Imbalance between TNFα and progranulin contributes to memory impairment and anxiety in sleep-deprived mice

**DOI:** 10.1038/srep43594

**Published:** 2017-03-16

**Authors:** Kun Zhang, Yu-jiao Li, Dan Feng, Peng Zhang, Ya-tao Wang, Xiang Li, Shui-bing Liu, Yu-mei Wu, Ming-gao Zhao

**Affiliations:** 1 Department of Pharmacology, School of Pharmacy, Fourth Military Medical University, Xi’an, 710032, China; 2 Precision Pharmacy & Drug Development Center, Tangdu Hospital, Fourth Military Medical University, Xi’an, 710038, China; 3 Department of Radiology, Tangdu Hospital, Fourth Military Medical University, Xi’an, 710038, China; 4 Department of Neurobiology, Capital Medical University, Beijing, 100069, China

## Abstract

Sleep disorder is becoming a widespread problem in current society, and is associated with impaired cognition and emotional disorders. Progranulin (PGRN), also known as granulin epithelin precursor, promotes neurite outgrowth and cell survival, and is encoded by the *GRN* gene. It is a tumor necrosis factor α receptor (TNFR) ligand which is implicated in many central nervous system diseases. However, the role PGRN in sleep disorder remains unclear. In the present study, we found that sleep deprivation (S-DEP) impaired the memory and produced thigmotaxis/anxiety-like behaviors in mice. S-DEP increased the levels of TNFα but decreased PGRN levels in the hippocampus. The intracerebroventricular (ICV) injection of PGRN or intraperitoneal injection of TNFα synthesis blocker thalidomide (25 mg/kg), prevented the memory impairment and anxiety behaviors induced by S-DEP. PGRN treatment also restored dendritic spine density in the hippocampus CA1 region and neurogenesis in hippocampus dentate gyrus (DG). These results indicate that an imbalance between TNFα and PGRN contributes to memory impairment and thigmotaxis/anxiety caused by sleep deprivation.

Sleep is critical for learning and memory, and sleep deprivation (S-DEP) is detrimental to learning, brain maturation, and waking consciousness^1^,^2^. The hippocampus appears to be more vulnerable to S-DEP than other brain areas^3^. Sleep-deprived mice exhibit memory impairments in hippocampus-dependent tasks, but not in hippocampus-independent tasks^4^. One explanation for hippocampus-dependent memory impairments is a lack of long-term potentiation (LTP) induction in the hippocampus CA1 region in S-DEP mice^5^.

The connection between S-DEP and memory impairment is not yet fully understood. Inflammation may play a crucial role in the relationship between sleep and cognition^6^. S-DEP impairs the physiological and behavioral development through the dysregulation of microglial pro- and anti-inflammatory cytokines^7^, such as tumor necrosis factor α (TNFα)^8^. S-DEP increases TNFα mRNA in the somatosensory cortex, frontal cortex, and basal forebrain brain^9^. Meanwhile, the serum and cortex TNFα levels are also increased when mice through the dysregulation of microglial pro- and anti-inflammatory cytokines^7^,^8^. However, the reason behind the TNFα up-regulation in S-DEP mice and its mechanism in S-DEP-induced memory impairments remain unknown.

TNFα receptor (TNFR) is highly expressed in the central nervous system (CNS) and plays important roles in CNS physiologies and pathologies. TNFα is a member of the type II transmembrane protein super-family, and is expressed in a full-length membrane-bound form (mTNF-α), which can be cleaved by the TNFα converting enzyme to release the soluble peptide form of TNFα (sTNFα)^10^. TNFR1 can bind to either soluble TNFα or transmembrane TNFα, with a preference for soluble TNFα, and activation of TNFR1 triggers a complex apoptotic pathway. In contrast, TNFR2 is preferentially activated by transmembrane TNFα and protects neurons against excitotoxicity^11^. In the CNS, the activation of TNFR1 is associated with alpha-amino-3-hydroxy-5-methyl-4-isoxazole-propionic acid (AMPA) receptor trafficking, excitability, and seizure susceptibility^12^. TNFα also plays a role in synaptic scaling and cognitive development^13^. Thus, a proper level of TNFα is required for normal brain function.

Progranulin (PGRN), also known as granulin epithelin precursor, is a PC cell (highly tumorigenic murine mesenchymal teratoma cells) derived growth factor encoded by the *GRN* gene with seven granulin repeats^14^. PGRN is a competitive inhibitor of TNFα which binds to TNFRs and prevents TNFα from interacting with TNFRs. Thus, PGRN could be used to treat inflammatory arthritis^15^,^16^. PGRN suppresses neutrophil chemotaxis caused by TNFα release from brain endothelial cells after cerebral ischemia-reperfusion^17^ and inhibits the expression of pro-inflammatory cytokines (TNFα, IL-1β, and IL-6) through TNFR2^18^,^19^. Despite of the anti-inflammatory effects, PGRN also promotes neurite outgrowth and cell survival in the CNS^20^,^21^. The down-regulation of PGRN decreases synapse density in primary hippocampal cultures^22^. PGRN expression is increased in activated microglia in neurodegenerative diseases including Creutzfeldt-Jakob disease, motor neuron disease, and Alzheimer’s disease^23^. Thus, PGRN is a critical regulator of TNFα function and the ratio of TNFα and PGRN requires further research attention^24^. However, the exact role of TNFα/PGRN balance in S-DEP-induced memory impairment remains unclear.

In this study, we found that S-DEP (72 h) induced long term memory impairment and anxiety-like behaviors in mice. In the hippocampus of S-DEP mice, TNFα protein level increased, whereas PGRN level decreased. Treatment with exogenous PGRN or inhibitor of TNFα (thalidomide) reversed the long term memory impairment and anxiety-like behaviors. Meanwhile, treatment with PGRN suppressed TNFα signaling hyperactivity and dendritic spine loss as well as restored neurogenesis in the dentate gyrus (DG) of the hippocampus in S-DEP mice. These findings suggest that down-regulation of PGRN plays a critical role in S-DEP-induced cognitive impairment.

## Results

### Sleep deprivation induces memory impairment and anxiety-like behaviors

**Figure 1:**
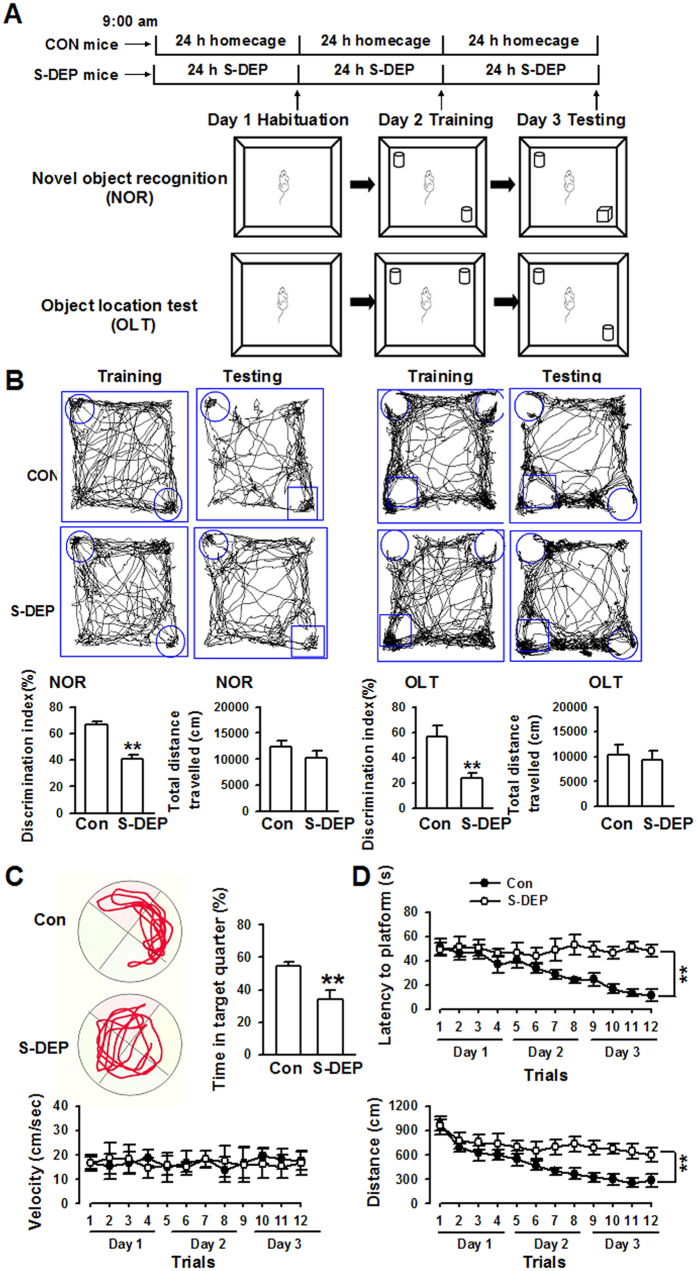
Sleep deprivation induced memory impairment. (**A**) Schematic of the novel object recognition test. (**B**) Left: sample traces of locomotor activity in the novel object recognition test for control (Con) and S-DEP mice respectively, preference index toward a novel object, and total distance travelled were summarized. Right: sample traces of locomotor activity in the object location test for control (Con) and S-DEP mice respectively, preference index toward a novel location and total distance travelled were summarized. (**C**) Left: Representative swimming paths in the water maze during the probe trial on day 3. Right: Percentage of time spent in the target quadrant of the water maze. (**D**) Latency to located platform position, velocity, and travelled distance during the learning phase of the water maze task. n = 10 per group; unpaired student t-test or repeated measures ANOVA, *p < 0.05 versus control mice.

S-DEP has been found to induce hippocampus-dependent cognitive impairment in rodents^25^. In the current study, mice were deprived of sleep for 72 h, followed by behavioral testing 24 h later (Fig. 1A). S-DEP significantly decreased the amounts of time spent interacting with the novel object, indicating that S-DEP impaired the memory of mice (*p* < 0.01, student t-test, Fig. 1B). Similarly, S-DEP mice showed impairments in novel location test, which is used to measure the ability of animals to remember the location of objects (*p* < 0.01, student t-test, Fig. 1B). To exclude the interference of locomotor activity, total distance traveled was analyzed. There was no difference between the S-DEP mice and control mice in the novel object and novel location tests. In Morris water maze test, the time in the target quadrant was significantly decreased in the S-DEP mice during the probe trials (*p* < 0.01, student t-test, Fig. 1C). The escape latencies to the platform and total swimming distance were markedly increased in the S-DEP mice during the learning trials (*p* < 0.01, repeated-measures analysis of variance, Fig. 1D). However, the swimming velocity was similar between the mice, indicating the intact locomotor activity of S-DEP mice.

**Figure 2:**
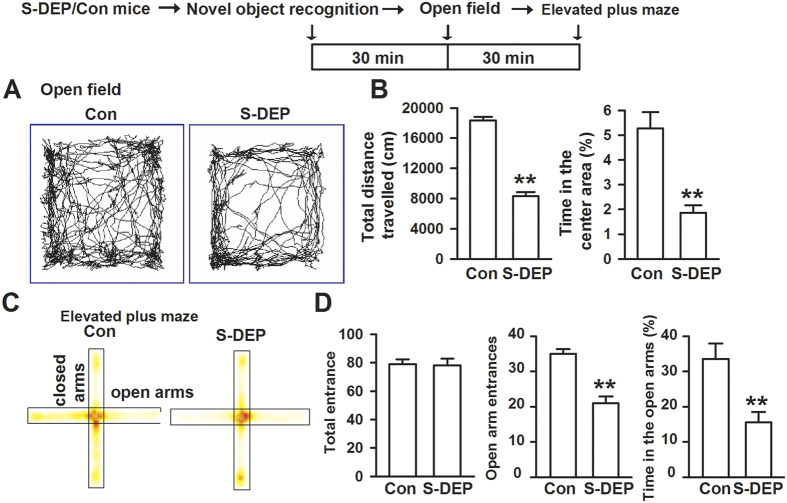
Sleep deprivation induced anxiety-like behaviors. Different tests were performed with the 30 min interval. (**A**) Sample traces of locomotor activity in the open field test. (**B**) S-DEP significantly reduced the total distance traveled and time spent in the center area. n = 10 mice per group. (**C**) Sample traces of locomotors activity in the elevated plus maze test. (**D**) S-DEP significantly reduced the entrance to open arms and time spent in the open arms. n = 10 mice per group; unpaired student t-test, **p < 0.01 versus control group.

Open field test and EPM were used to test thigmotaxis/anxiety-like behaviors. In the Open field test, both the total distance traveled and the time spent in the center area was decreased in S-DEP mice (*p* < 0.01, student t-test, Fig. 2A and B). In the EPM test, no difference of total entrance to the open and closed arms was found between control and S-DEP mice. However, the number of entrance to the open arms and the time spent in open arms were notably decreased in the S-DEP mice (*p* < 0.01, student t-test, Fig. 2C and D). These results indicate that S-DEP induces the learning and memory deficits and anxiety-like behaviors.

### Sleep deprivation induces an imbalance between TNFα and PGRN

**Figure 3:**
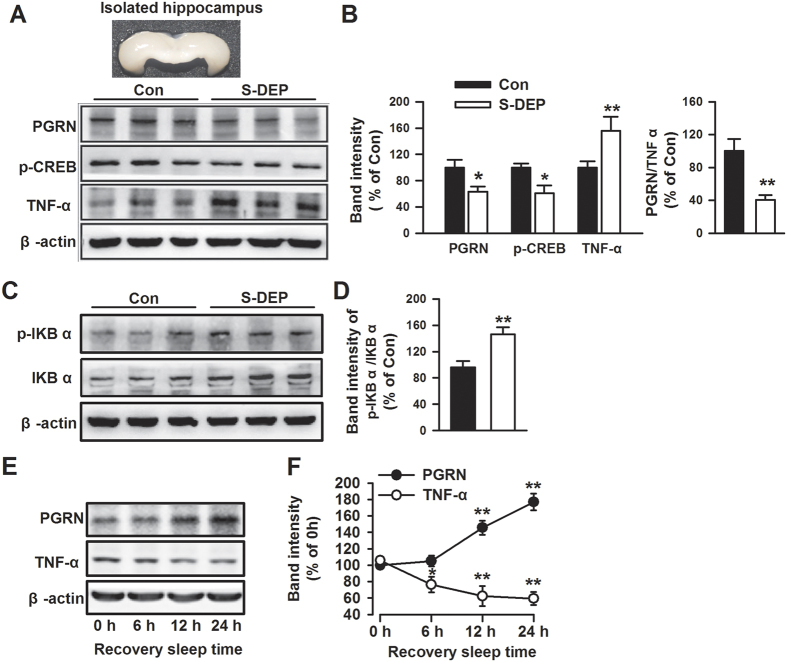
Effects of sleep deprivation on the levels of TNFα and PGRN. (**A**) Western-blot samples of PGRN, p-CREB, and TNFα. (**B**) Band intensities were quantified as percentage of values from Control mice hippocampus. n = 6 mice per group; unpaired student t-test, **p < 0.01 versus control mice. (**C**) Western-blot samples of p-IκBα and IκBα. (**D**) Band intensities were quantified as percentage of values from control mice hippocampus. n = 6 mice per group; unpaired student t-test, *p < 0.05, **p < 0.01 versus control mice. (**E**) Western-blot samples of PGRN and TNFα after recovery sleep. (**F**) Band intensities were quantified as percentage of values from 0 hour recovery sleep mice. n = 4 mice per group; unpaired student t-test, **p < 0.01 versus 0 hour recovery sleep mice.

Next, alterations of TNFα and PGRN were detected in the hippocampus of S-DEP animals. We found that S-DEP induced a significant increase in TNFα level and a decrease in PGRN level in the hippocampus (*p* < 0.05 and *p* < 0.01 respectively, student t-test, Fig. 3A and B). The level of phosphorylated cAMP response element binding protein (p-CREB) is closely linked with learning and memory^26^,^27^. NF-κB is one of the major down-stream targets of TNFα signaling^28^. As shown by the results, S-DEP significantly decreased the levels of p-CREB (*p* < 0.05, student t-test, Fig. 3B) and increased the levels of phosphorylated IκB and the ratio of p-IκBα/IκBα (*p* < 0.01, student t-test, Fig. 3C and D). To confirm whether PGRN and TNFα changes were produced by S-DEP, recovery sleep was assigned to the S-DEP mice. As shown in Fig. 3E and F, 24 h of recovery sleep could effectively reverse the alterations of PGRN and TNFα in S-DEP mice (*p* < 0.01, student t-test). Taken together, these findings suggest that S-DEP induces the activation of the TNFα and the inhibition of PGRN in the hippocampus.

### Blocking the TNFα pathway attenuates S-DEP-induced memory impairment

**Figure 4:**
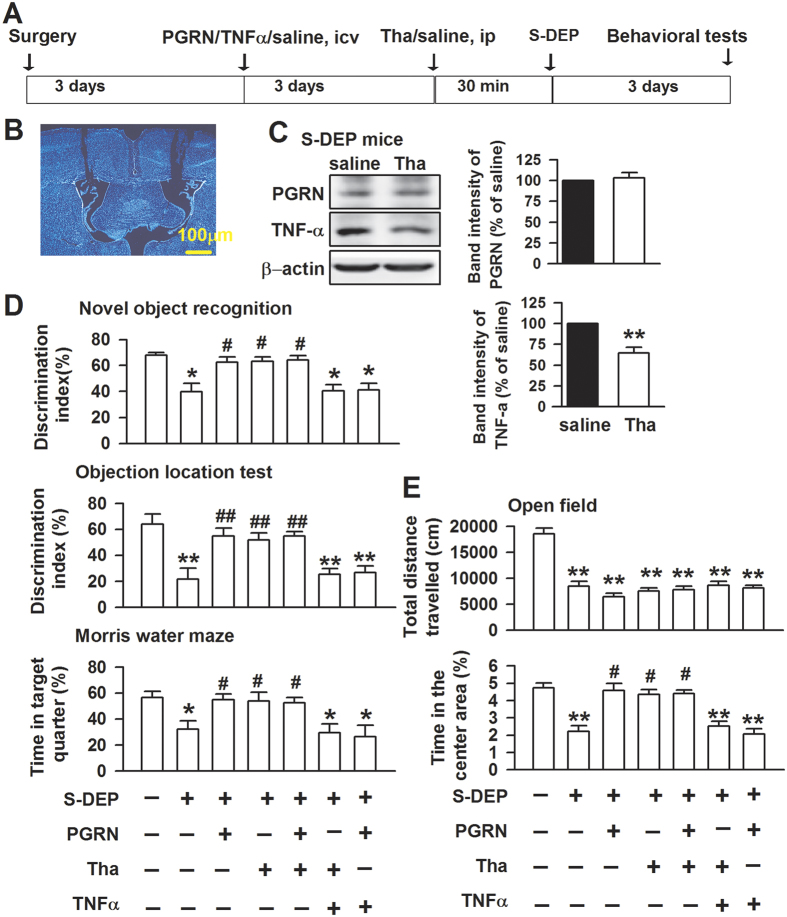
Blocking TNFα pathway attenuated memory impairment and anxiety-like behaviors. (**A**) Schematic of experimental procedures. (**B**) Sample of PGRN intraventricular injection (ICV) site. (**C**) Western-blot samples of PGRN and TNFα and band intensities after thalidomide (Tha) treatment in S-DEP mice. (**D**) Preference index of control, S-DEP, PGRN (ICV, 5 ng), and Tha (ip, 25 mg/kg) treated mice in novel object recognition test. (**E**) Locomotors activities and time spent in the center area of control, S-DEP, PGRN (ICV, 5 ng), and Tha (ip, 25 mg/kg) treated mice in open field test. n = 6 mice per group; one-way ANOVA with LSD test, *p < 0.05, **p < 0.01 versus control mice, ^#^*p* < 0.05 versus saline treated S-DEP mice.

TNFR1 knockout mice show less sleep during the light period, indicating that TNFα is an important sleep regulator^29^. In the current study, S-DEP induced higher levels of TNFα in the hippocampus (Fig. 3B). Next, we detected the behaviors in the S-DEP mice by interfering with the TNFα synthesis or treatment of mice with exogenous TNFα and PGRN (Fig. 4A and B). The intraperitoneal (ip) injection of thalidomide (25 mg/kg), a TNFα synthesis blocker, effectively reduced the TNFα level in the hippocampus (*p* < 0.01, student t-test, Fig. 4C). The ICV injection of PGRN (5 ng) 3 days before S-DEP alleviated S-DEP-induced memory impairment (*F*_(6,35)_ = 8.891, *p* = 0.000, LSD test; Fig. 4D) and thigmotaxis/anxiety-like behaviors (total travel distance: *F*_(6,35)_ = 32.449, *p* = 0.000, LSD test; time in the central areas: *F*_(6,35)_ = 16.063, *p* = 0.000, LSD test; Fig. 4E). Similarly, thalidomide attenuated the memory impairment and thigmotaxis/anxiety-like behaviors induced by S-DEP. However, combining PGRN with thalidomide did not cause additional improvement in the performance of S-DEP mice. Additionally, the ameliorations of memory impairment and anxiety by PGRN and thalidomide could be blocked by TNFα ICV injection, thereby indicating the critical role of PGRN/TNFα ratio in the behaviors. These results suggest that PGRN and thalidomide restore the memory and thigmotaxis/anxiety-like behaviors through a TNFα-dependent manner.

### PGRN blocks the activation of TNFα signaling by S-DEP

**Figure 5:**
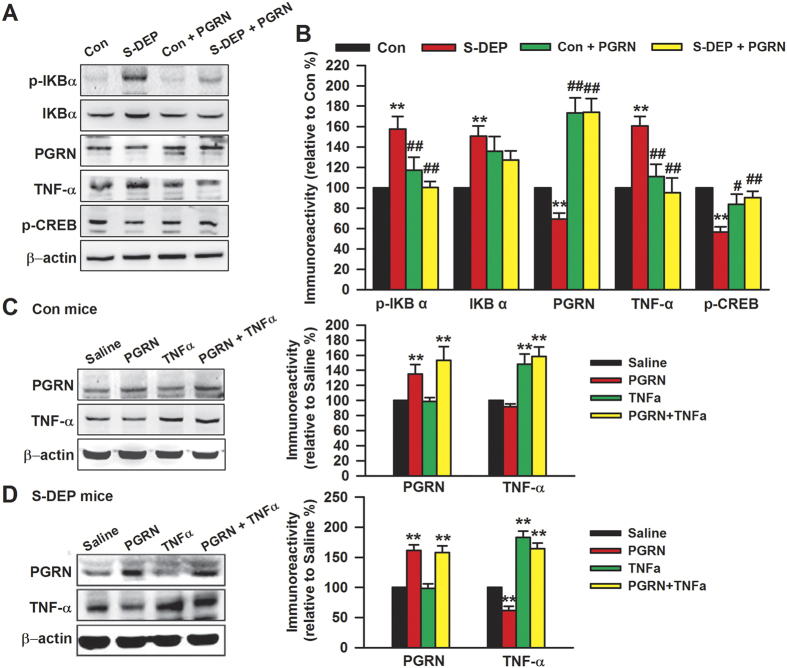
PGRN antagonized the TNFα signaling. (**A**) Mice were sacrificed after behavior tests. The levels of p-IκBα, IκBα, PGRN, TNFα, and p-CREB in hippocampus were detected by Western blot. (**B**) Band intensities were quantified as percentage of values from control mice. n = 6 mice per group, Two-way ANOVA with Bonferroni’s Multiple Comparison Test; *p < 0.05, **p < 0.01 versus control mice, ^#^*p* < 0.05, ^##^*p* < 0.01 versus saline treated S-DEP mice. Levels of PGRN and TNFα in hippocampus were detected after exogenous PGRN and TNFα treatment in control mice (**C**) and S-DEP (**D**) mice. n = 6 mice per group; one-way ANOVA with LSD test, *p < 0.05, **p < 0.01 versus control mice, ^#^*p* < 0.05, ^##^*p* < 0.01 versus saline treated S-DEP mice.

To further verify the role of the TNFα pathway in PGRN activity, we measured the levels of p-IκBα, IκBα, PGRN, TNFα, and p-CREB, which are involved in TNFα signaling and memory consolidation in the hippocampus (Fig. 5A). ICV injection of PGRN significantly increased the levels of PGRN and p-CREB in the hippocampus of S-DEP mice (F_(3,20)_ = 22.64, *p* < 0.01, and F_(3,20)_ = 6.32, *p* < 0.05, respectively, Fig. 5B). Meanwhile, the activation of p-IκBα and TNFα caused by S-DEP was reversed in mice injected with PGRN (F_(3,20)_ = 16.31, p < 0.01, and F_(3,20)_ = 18.29, *p* < 0.01 respectively). To validate the interaction between PGRN and TNFα, levels of PGRN and TNFα in the hippocampus were detected after exogenous PGRN and TNFα treatment in control mice and S-DEP mice. As shown in Fig. 5C and D, PGRN decreased TNFα level in S-DEP mice (*F*_(3,20)_ = 38.544, *p* = 0.000, LSD test; Fig. 5D), but not in control mice; moreover, TNFα failed to affect PGRN levels both in control and S-DEP mice. These results further imply that PGRN pretreatment prevents S-DEP-induced memory and emotional impairments through regulation of TNFα signaling.

### PGRN restores dendritic spine density and neurogenesis in hippocampus

**Figure 6:**
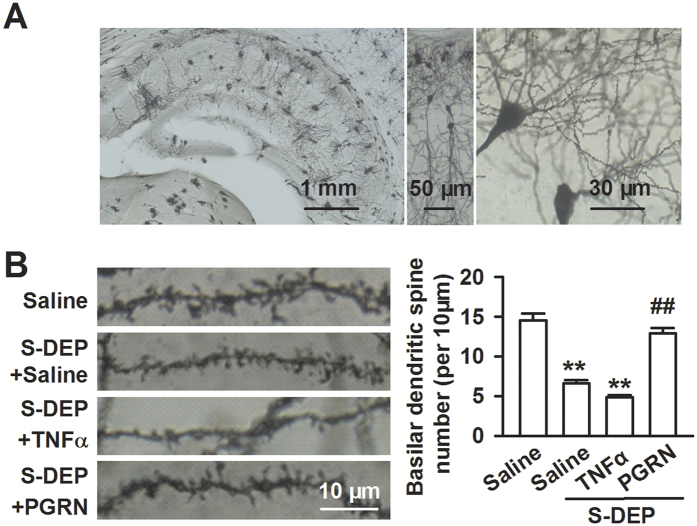
PGRN treatment reversed the spine density in CA1 area. (**A**) Golgi-Cox-staining of CA1 pyramidal neurons for spine counting. (**B**) Left: Representative images of basilar dendrites from control, saline (ICV, 5 μl) treated S-DEP, TNFα treated (ICV, 10 ng/5 μl) S-DEP, and PGRN treated (ICV, 5 ng/5 μl) S-DEP mice. Right: Summary of spine counts from basilar dendrites. n = 40 neurons/4 mice per group; two-way ANOVA with LSD test, **p < 0.01 versus control mice, ^##^*p* < 0.01 versus saline treated S-DEP mice.

Dendritic spines are the basic structural units that underlie the learning and memory formation^30^,^31^. Spines lose in the hippocampus region has been attributed to depression^32^, Alzheimer disease^33^, and Rett syndrome^34^; it has also been observed in S-DEP mice^35^. Given the role of PGRN and TNFα signaling in S-DEP-induced memory impairment, we predicted that manipulation of this signaling would affect spine density (Fig. 6A). Consistent with this hypothesis, the ICV injection of PGRN significantly increased spine density in CA1 in S-DEP mice (*F*_(3,12)_ = 56.41, *p* = 0.000, LSD test; Fig. 6B).

**Figure 7:**
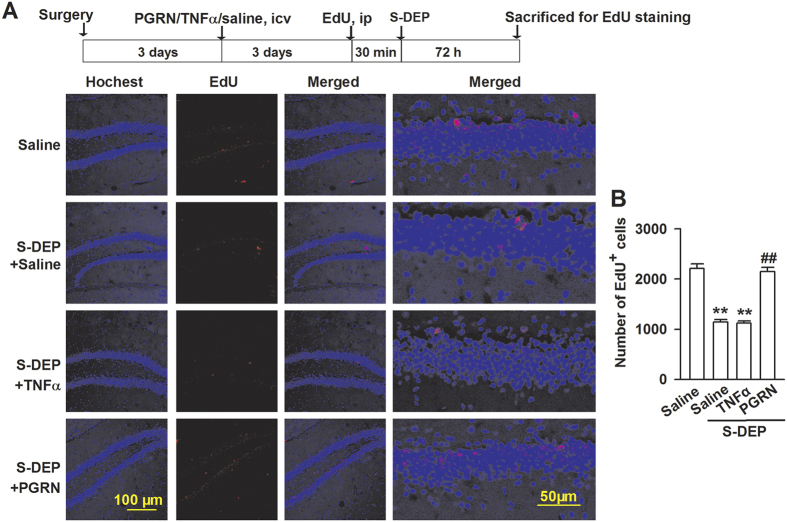
PGRN increased neurogenesis in S-DEP mice. (**A**) Representative micrograph of double staining to identify newborn neurons (EdU/red) in the dentate gyrus of the hippocampus. Left: scale bar = 100 μm; Right: scale bar = 50 μm. (**B**) Number of EdU positive neurons in the dentate gyrus subgranular zone from control, saline (ICV, 5 μl) treated, TNFα treated (ICV, 10 ng/5 μl), and PGRN treated (ICV, 5 ng/5 μl) S-DEP mice. n = 3 mice per group; one-way ANOVA with LSD test, **p < 0.01 versus saline treated control mice, ^##^*p* < 0.01 versus saline treated S-DEP mice.

Neurogenesis is involved in proliferation, differentiation, maturation, migration, survival, and functional integration of new neurons into the existing hippocampal network^36^. The total REM phase in S-DEP produces an 82% decrease in the percentage of hippocampus Brdu^+^ neurons^37^. Thus, the number of neurogenesis is used to evaluate the impairment by S-DEP. In current study, S-DEP significantly decreased neurogenesis in subgranular zone of DG, as shown by the EdU labeling of actively dividing neurons (*F*_(3,8)_ = 8.482, *p* = 0.007, LSD test; Fig. 7A and B). The ICV injection of PGRN significantly restored the number of EdU-positive neurons in S-DEP mice. TNFα treatment did not further decrease the number of EdU positive neurons in S-DEP mice, suggesting that PGRN induces neurogenesis in DG of S-DEP mice.

## Discussion

In the present study, we find that S-DEP induces the over-expression of TNFα, but down-regulates PGRN expression in the hippocampus. Accordingly, S-DEP induces behavioral abnormalities, including anxiety-like behaviors and memory impairments. The ICV injection of PGRN prevents these behavioral abnormalities and the activation of TNFα signaling in the hippocampus. Furthermore, PGRN treatment reverses the dendritic spine loss and down-regulation of neurogenesis in the DG caused by sleep deprivation. Thus, this study provides evidence that PGRN is a potential candidate for the treatment of S-DEP-induced cognitive impairment and emotional disorders.

Sleep is critical for learning and memory consolidation^38^. For declarative memory, slow wave sleep has a beneficial effect on the consolidation of memories acquired during preceding wakefulness^3^,^38^. Sleep/wake cycles are controlled by the super chiasmic nucleus in the hypothalamus, but can be disrupted by diseases of the nervous system, causing sleep disorders. In the current study, modified multiple platform model disrupts 90–95% of REM sleep of mice, but it also affects non-REM sleep^39^, although the disruption of non-REM sleep may be inconsequential to our finding. Evidence suggests that REM sleep is more likely than non-REM sleep to play a critical role in the formation and consolidation of memory^40^,^41^. According to Gina Poe’s more recent work, REM S-DEP could impair both emotional and contextual memories^42^,^43^. In present study, novel object recognition, novel location test, and Morris Maze were used to detect the learning and memory ability of the mice. S-DEP induced memory impairments. Interestingly, we found that the total distance traveled in the object recognition task and novel location test had no difference between the S-DEP mice and control mice. This could exclude the possible lower function of locomotor activity of S-DEP mice in object recognition tasks. However, hypoactivity was found in the open field test. This may be the objects put in the novel object recognition, novel location testing box, and mice had the instincts to explore these objects.

PGRN has been reported to play an important role in maintaining normal spine density and morphology^44^. Our finding of decreased PGRN could partially explain the memory impairment in S-DEP mice. However, how PGRN and TNFα interact each other in the physiological and S-DEP conditions remains unknown. It is reported that sleep disruption induces sleepiness and cognitive dysfunction via the TNFα pathway^45^. This finding is consistent with our findings that S-DEP induces a higher level of TNFα in the hippocampus.

In the current study, S-DEP decreased the levels of p-CREB, a protein that is closely linked with learning and memory^26^,^27^. This finding is consistent with the memory impairment we observed in the NOR test. In the same time, S-DEP increased the levels of phosphorylated IκB and the ratio of p-IκBα/IκBαNF-κB, the major down-stream targets of TNFα signaling^28^. Taken together, these findings suggest that S-DEP induces the activation of the TNFα signaling pathway.

TNFα-TNFR signaling has garnered great attention owing to its important role in CNS pathologies. TNFα activates the membrane-bound TNF receptors TNFR1 and TNFR2. Of the two, TNFR1 can bind to either soluble TNFα or transmembrane TNFα, with a preference for soluble TNFα, and the activation of this receptor triggers a complex apoptotic pathway. In contrast, TNFR2 is preferentially activated by transmembrane TNFα and protects neurons against excitotoxicity^11^. In the CNS, activation of TNFR1 is associated with AMPA trafficking, enhanced excitability, and seizure susceptibility^12^. TNFα also plays a role in synaptic scaling and cognitive development^13^. Thus, a properly titrated level of TNFα is required for normal brain function. Our results indicate that higher levels of TNFα induced by S-DEP are harmful to cognition. Thalidomide is widely used to inhibit TNFα synthesis and attenuates the neuronal loss and cognitive impairments^46^,^47^. Thalidomide lowers TNF-α protein levels by enhancing the degradation of TNFα mRNA^48^. The amounts of interleukin-1β, interleukin-6, and granulocyte/macrophage colony-stimulating factor produced by monocytes remain unaltered. Hence, the selectivity of this drug may be useful in determining the role of TNFα *in vivo* and modulating its toxic effects in a clinical setting^49^. In the present study, we found that the systemic treatment of thalidomide reversed the memory impairment and thigmotaxis/anxiety-like behaviors induced by sleep deprivation, thus confirming the functional role of the TNFα in the sleep deprivation.

PGRN can bind to TNFR1 and TNFR2 receptors and act as an anti-inflammatory cytokine^15^; it also suppresses inflammation by targeting TNFα signaling^50^. Compared with TNFα, PGRN shows a much higher affinity to TNFR2^15^. The current study shows that elevating PGRN in the hippocampus is an effective way to improve the memory impairment caused by sleep deprivation, thereby extending our understanding of PGRN function. PGRN suppressed TNFα expression in S-DEP mice but not in control mice. However, TNFα did not affect PGRN expression in both control and S-DEP mice. It seemed that levels of PGRN and TNFα were regulated independently. At the same time, the ICV injection of PGRN restored the S-DEP-induced decreases of spine density in the CA1 region and neurogenesis in the DG, which may be the cellular mechanisms underlying the improvement of cognitive function. This suggests that both CA1 and DG is involved in the learning and memory deficit induced by SDEP. However, the downstream pathway of PGRN requires further study. TNFα is known to perturb blood-brain barrier integrity and promote brain edema formation^51^. Thus, it cannot exclude that the increased TNFα may cause the cognitive impairment and anxiety-like behavior by directly destroying the blood-brain barrier integrity.

In summary, our research indicates that restoring the balance between TNFα/PGRN in the brain can ameliorate memory impairment caused by sleep deprivation. It will be helpful to the development of drugs for treatment of insomnia-induced cognitive impairment.

## Materials and Methods

Study protocol was approved by the Ethics Committee of Fourth Military Medical University. All procedures were performed in accordance with our Institutional Guidelines for Animal Research and the investigation conformed to the Guide for the Care and Use of Laboratory Animals published by the US National Institutes of Health (NIH Publication No. 85-23, revised 2011).

### Animals

Eight to ten week-old C57BL/6 J male mice were used in the experiments. The mice were obtained from the Laboratory Animal Center of the Fourth Military Medical University. The animals were housed in plastic boxes in groups of 4 with food and water available in a colony room with controlled temperature (24 ± 2 °C), humidity (50–60%), and a luminous intensity of 100 lx with a light cycle from 8:00 AM to 8:00 PM. Mice were allowed to adapt to laboratory conditions for at least 1 week before the procedure. All behavioral tests were performed during 9 AM to 12 AM on the designated day of experiment.

### Induction of S-DEP

S-DEP was induced as described previously^52^. Briefly, mice were placed on the platforms (2.5 cm in diameter, 1 cm above the water surface) in a water-filled tank with 12 platforms. The platforms were spaced at a distance of 5 cm so that mice could move freely from one platform to another. The mice had free access to water and food. When the animal enters the rapid eye movement (REM) phase of sleep, it falls into the water due to the muscle atonia and the small platform size, and wakes up. The duration of REM deprivation (72 h) was determined on the basis of previous studies in which mice that were deprived of REM for this period of time presented memory deficits in avoidance tasks^53^. During the sleep deprivation period (72 h), the temperature (23 ± 1 °C) and light/dark cycle were both maintained under controlled conditions. This method deprived 95% REM sleep and effectively decreased the time spent in slow wave sleep by 31%^54^. The control group mice did not experience S-DEP, but were housed in their homecages.

### Novel object recognition (NOR)

The NOR test was performed as described previously^55^. Inanimate, acrylic, and blue colored objects were used in this test. The cylinder and cube share the same volume. The apparatus consisted of a container (25 cm length, 25 cm width, and 20 cm height) made of polyvinyl chloride, and was put in a sound proof box with a digital camera on the roof. Two row LED tubes were fixed above two sides of the closed container. S-DEP mice were placed in the behavioral chamber to acclimate to the new environment (Day 1). Each mouse was individually habituated to the apparatus for 10 min in the absence of objects (habituation trial). The next day (Day 2), the mice were placed in the apparatus, and 2 identical cylinders were placed in a symmetrical corner of the box (training trial). On the test day (Day 3), one cylinder was replaced with a cube of similar volume (testing trial) (Fig. 1A). In training and testing trials, the mice were allowed to explore freely for 10 min, and the time spent exploring each object was recorded. In the object-place recognition test, three objects were used in the training and testing phases. In the test phase, two objects that were identical to the ones in the training phase were placed in the container, but one was in the same location as the training phase (familiar object-place) and the other was moved to a different location (novel object-place). After each phase the floor and the objects were wiped with 70% ethanol. The video was recorded by a camera connected with the computer. Time spent exploring each object during the training and test phase were scored by software (Shanghai Jiliang, China). Interaction parameters were defined as contact with the object (tail only excluded) or facing the object (distance < 2 cm). The proportion of the exploration time exploring the novel object was defined as the “object preference index” expressed by the ratio of TN/(TF + TN) (TF: time spent exploring the familiar object; TN: time spent exploring the novel object).

#### Morris water maze

Morris water maze task was conducted in a white circular tank, 120 cm in diameter and 50 cm deep, filled with 20 cm of white opaque water (liquid milk) that was maintained at 24 °C. A pneumatically controlled escape platform 10 cm in diameter was located at a fixed position midway between the center and the wall. The platform was usually 0.5 cm below the water surface. During spatial learning, mice were trained for four trials per day for three consecutive days. To promote invariant cue representations forming during training, distinct start locations were used for each trial: north-western (NW), northern (N), eastern (E), or south-eastern (SE). These distal positions relative to the hidden platform were chosen to minimise mice randomly achieving the escape goal at the beginning of navigation. The start location trial assignments were randomized across the three days of training. Training in the Morris water maze consisted of eight blocks of four trials. The mice were given two blocks of trials per day with an interblock interval of 3 h. The start position for each trial was randomly drawn from four possible positions. During each trial, mice were allowed to search for the escape location for a maximum of 60 s. If mice did not find the hidden platform location during this period mice were gently guided to it. After finding the platform, mice were left on it for 30 s^56^. Retention tests, or probe trials, with the escape platform submerged to the bottom of the pool, were conducted on days 3. The position of the mice was tracked and stored at 10 Hz by a video tracking system (Any-Maze, Stoelting, IL, USA).

### Locomotor activity test

The locomotor activity test was conducted as described previously^57^. It was carried out in the Open field, a square arena (30 cm × 30 cm × 30 cm) with clear Plexiglas walls and floor, and placed inside an isolation chamber with dim illumination and a fan. Mice were placed in the center of the box and allowed to freely explore for a 15 min period. Mice were videotaped using a camera fixed above the floor and analyzed with a video-tracking system (Shanghai Jiliang, China).

### Elevated plus maze

The Elevated plus maze (EPM) was conducted as described previously^58^. The apparatus (DigBehv-EPMG, Shanghai Jiliang, China) comprised of two open arms (25 cm × 8 cm × 0.5 cm) and two closed arms (25 cm × 8 cm × 12 cm) that extended from a common central platform (8 cm × 8 cm). The apparatus is elevated to a height of 50 cm above the floor. Mice were allowed to habituate to the testing room for 2 days before the test, and pretreated with gentle handling two times per day to minimize anxiety. For each test, individual animal was placed in the center square, facing an open arm, and allowed to move freely for 5 min. Mice were videotaped using a camera fixed above the maze and analyzed with a video-tracking system. The entrance is defined as all four paws placed inside an arm. The number of entrance and time spent in each arm were recorded.

### Western blot analysis

Six mice in each group were sacrificed for western blot immediately after all behaviors tests. Another 18 mice in each group were sacrificed 6, 12, and 24 hours later after sleep deprivation respectively. Western blot analysis was performed as described previously^59^. After behavior tests, mice were exposed to isoflurane vapors for <1 min and then rapidly decapitated. Brain was carefully removed and immediately placed on ice (<2 min relative to initial handling). Hippocampus was removed with a microscissor and frozen in liquid nitrogen, and stored at −80 °C until further analysis. Snap-frozen hippocampus was homogenized by ultrasonic wave in ice-cold RIPA lysis buffer. The homogenate of was separated by centrifugation at 14,000 g for 15 minutes, and the supernatant containing total cellular proteins was collected. The protein concentration was determined by microplate BCA protein assay kit (Pierce Biotechnology, Rockford, IL, USA) and samples were then subjected to western blotting analysis. Equal amounts of protein (50 μg) from the hippocampus were separated and electro transferred onto PDVF membranes (Millipore, Billerica, MA. USA), which were probed with antibodies against p-CREB (dilution ratio, 1:1000, Cell Signaling Technology, Danvers, MA, USA), p-IκBα (dilution ratio, 1:1000, Sangon Biotech, Shanghai, China), IκBα (dilution ratio, 1:1000, Sangon Biotech), TNFα (dilution ratio, 1:1000, Cell Signaling Technology), PGRN (0.5 μg/ml, R&D system, Minneapolis, MN, USA), β-actin (dilution ratio, 1:10000, Sigma, St. Louis, MO, USA) as the loading control. For data quantification, band intensity of each blot was calculated as ratio relative to β-actin. The intensity ratio of the control group was set as 100%, and the intensity of other treatment groups were expressed as percentage to the control group. The membranes were incubated with horseradish peroxidase-conjugated secondary antibodies (anti-rabbit/anti-mouse IgG for the primary antibodies), and blots were developed using either standard or enhanced chemiluminescence detection (Millipore, USA or Genshare Biological, China) and imaged using a Tanon imaging system (Tanon 4200, China).

### Stereotactic injection

For the stereotactic injection, mice were anesthetized with pentobarbital sodium (30 mg/kg, i.p.) and thereafter were placed on a stereotaxic apparatus. A stainless steel cannula was placed with the tip positioned at the coordinates (anterior–posterior −0.5 mm, lateral 2 mm, and horizontal 2.2 mm) as reported previously^60^. The cannula was fixed with jeweler acrylic cement, which was also used to cover the small surface of the skull. After surgery, mice were maintained in single cages and recovered from surgery for 3 days. Mice received intracerebroventricular (ICV) injections of recombinant PGRN (5 ng/5 μl in 0.9% saline; Sino Biological Inc., Beijing, China), TNFα (10 ng/5 μl in 0.9% saline; Sino Biological Inc.), or 5 μl 0.9% saline by a polyethylene tube to a 10 μL Hamilton syringe at a rate of 1 μl/min 3 days before sleep deprivation. After behavior tests, mice were scarified and the proteins were extracted from hippocampus for western blot analysis.

### Golgi-Cox staining and spine density analysis

Golgi staining was performed as described previously^61^. Another group of mice (4 mice in each group) were carried out stereotactic injection for Golgi staining. As S-DEP inducing memory impairment and anxiety have confirmed, the S-DEP mice were used for Golgi-Cox staining after S-DEP without behavior tests. Briefly, brains were removed and immersed in 10 ml of Golgi-Cox solution (1% potassium dichromate, 1% mercuric chloride, 0.75% potassium chromate) and stored in a tightly sealed glass jar in the dark at room temperature for 12 days. Brains were sectioned (120 μm) using a vibratome. After gradient ethanol dehydration, hippocampus slices were mounted onto slides using neutral balsam and imaged on Olympus BX51 light microscope using DP-BSW software with a 100x/NA 1.4 oil immersion lens. Analyses were performed on basilar dendrites from DG CA1 neurons for total length of 100 μm. Spines from three dendrites of selected neuron were counted. Spine densities were measured by counting the number of spines of all types at intervals of 10 μm on dendrites and analyzed using ImageJ. Statistical analyses for spine count were done by pooling spine counts of all neurons according to different types and different treatments. Values from all neurons derived from the same animal were consolidated together and averaged. All analyses were conducted by an experimenter blind to treatment. Dendritic spines in 120 dendrites of 40 neurons from 4 mice pre group were counted.

### Staining for neurogenesis

To assess the number of new born neurons in the DG, 5-ethynyl-2′-deoxyuridine (EdU) staining was performed as previously^62^. Another 12 mice (3 mice in each group) were carried out stereotactic injection for EdU staining. EdU (100 μg) was administered intraperitoneally on the day before S-DEP. Mice were anaesthetized with ketamine and xylazine, and transcardially perfused with cold saline, followed by 4% cold paraformaldehyde (PFA) in PBS. Brains were removed and fixed in 4% PFA overnight at 4 °C, then cryo-protected in 30% sucrose and stored at 4 °C. Coronal hippocampus sections (10 μm) were cut by freezing microtome (CM1950, Leica, Germany), and mounted onto glass slides. EdU staining was conducted using Click-iT^®^ EdU Assay (AlexaFluor^®^ 555) (Invitrogen) according to the manufacturer’s protocol. Cell nucleuses were stained by Hoechst 33342. Sections were mounted onto slides using 50% glycerinum. The slides were observed under a confocal laser microscope (FV1000, Olympus, Tokyo). Photomicrographs were saved as TIF files without any manipulation, and quantitatively analyzed with the cell counter of ImageJ software. Counting EdU positive neuron was carried out as reported previously^63^. Briefly, every sixth section of the brain containing hippocampus was selected and immunolabelled with EdU and Hoechst 33342 in dentate gyrus subgranular zone. Total numbers of positive cells in all slices per animal were multiplied by six to estimate the number of cells per DG.

### Statistical analysis

All the results are expressed as the mean ± SEM. Statistical analyses of the data were performed using SPSS V8. Unpaired student t-test was used to test difference between the two groups. One-way or two-way ANOVA tests were used to test the difference among multiple groups followed by *post-hoc* testing (with Tukey-Kramer correction for multiple comparisons). The LSD test was used for One-way ANOVA tests. Newman–Keuls *post hoc* test was used for two-way ANOVA tests. In all cases, probability value of *p* < 0.05 was considered significant.

## Additional Information

**How to cite this article**: Zhang, K. *et al*. Imbalance between TNFα and progranulin contributes to memory impairment and anxiety in sleep-deprived mice. *Sci. Rep.* **7**, 43594; doi: 10.1038/srep43594 (2017).

**Publisher's note:** Springer Nature remains neutral with regard to jurisdictional claims in published maps and institutional affiliations.
